# Intracellular bacteria are common and taxonomically diverse in cultured and *in hospite* algal endosymbionts of coral reefs

**DOI:** 10.1038/s41396-021-00902-4

**Published:** 2021-02-08

**Authors:** Justin Maire, Sam K. Girvan, Sophie E. Barkla, Alexis Perez-Gonzalez, David J. Suggett, Linda L. Blackall, Madeleine J. H. van Oppen

**Affiliations:** 1 School of Biosciences, The University of Melbourne, Melbourne, VIC Australia; 2 Melbourne Cytometry Platform, Melbourne Dental School, The University of Melbourne, Melbourne, VIC Australia; 3 Climate Change Cluster, Faculty of Science, University of Technology Sydney, Sydney, NSW Australia; 4 Australian Institute of Marine Science, Townsville, QLD Australia

**Keywords:** Symbiosis, Microbiome, Marine microbiology

## Abstract

Corals house a variety of microorganisms which they depend on for their survival, including endosymbiotic dinoflagellates (Symbiodiniaceae) and bacteria. While cnidarian–microorganism interactions are widely studied, Symbiodiniaceae–bacteria interactions are only just beginning to receive attention. Here, we describe the localization and composition of the bacterial communities associated with cultures of 11 Symbiodiniaceae strains from nine species and six genera. Three-dimensional confocal laser scanning and electron microscopy revealed bacteria are present inside the Symbiodiniaceae cells as well as closely associated with their external cell surface. Bacterial pure cultures and 16S rRNA gene metabarcoding from Symbiodiniaceae cultures highlighted distinct and highly diverse bacterial communities occur intracellularly, closely associated with the Symbiodiniaceae outer cell surface and loosely associated (i.e., in the surrounding culture media). The intracellular bacteria are highly conserved across Symbiodiniaceae species, suggesting they may be involved in Symbiodiniaceae physiology. Our findings provide unique new insights into the biology of Symbiodiniaceae.

## Introduction

Virtually all eukaryotes associate with symbiotic bacteria [1–3], and unicellular algae are no exception. Algae–bacteria associations are widespread in the marine environment, including the pelagic zone [4, 5] and coral reefs [6]. Extensive interactions in the phycosphere, an extracellular boundary layer composed of algal exudates, such as fixed organic carbon, may attract and nurture bacteria [7, 8]. Moreover, bacteria have been observed inside many types of algae [9–13], indicating the existence of intracellular interactions. Bacteria are known to provide their algal hosts with vitamin B12 [14–17], increased iron bioavailability [18], carotenoids [19], and growth-promoting hormones [20], though the array of functions is likely much greater.

One major group of marine microalgae, the Symbiodiniaceae (Suessiales, Dinophyta) is well known for its obligate endosymbiosis with cnidarians, including reef-building corals and sea anemones [21, 22]. The Symbiodiniaceae are highly diverse, with nine described genera and three additional evolutionary lineages that currently lack formal nomenclature [23, 24], encompassing free-living species and species associated with a wide range of marine organisms, including cnidarians, mollusks, and protists [21]. By translocating photosynthate to the cnidarian tissues, Symbiodiniaceae provide cnidarian hosts with most of their carbon requirements [25–27]. This photosymbiosis allows corals to build the reef’s three-dimensional structure, thus providing habitat to many other reef-dwelling organisms. Coral reefs are rapidly declining globally because of increasingly frequent episodes of mass coral bleaching caused by heatwaves [28, 29]. Coral bleaching is the breakdown of the symbiosis between Symbiodiniaceae and coral hosts. It is thought to be the result of excess production of reactive oxygen species (ROS) by Symbiodiniaceae under excessive temperature and/or light exposure, and leads to coral starvation and mortality [30–32]. Thus, unlocking the ecology and physiology of Symbiodiniaceae is a priority in global efforts to mitigate coral bleaching and minimize the impacts of climate change on coral reefs.

Aside from diverse Symbiodiniaceae, cnidarians associate with abundant and diverse bacterial communities [22, 33, 34]. Specific bacteria have been shown to be involved in nutrient cycling within the coral holobiont [35–37], such as metabolic exchanges linked to the sulfur and nitrogen cycles between Symbiodiniaceae and bacteria [35, 38]. More recently, a bacterial strain closely related to *Muricauda* was shown to provide zeaxanthin to cultured Symbiodiniaceae (*Durusdinium* sp.), thereby enhancing its resistance to light and temperature stresses [19]. Hence, investigating the bacterial communities that naturally associate with Symbiodiniaceae is a key step toward a broader understanding of the impact of Symbiodiniaceae–bacteria interactions on cnidarian health.

To date, few studies have investigated the microbiome of Symbiodiniaceae [39–41]. Lawson et al. reported the composition of the bacterial communities associated with 18 Symbiodiniaceae cultures [40]. Communities were dominated by Alpha- and Gammaproteobacteria with *Labrenzia*, *Marinobacter*, and Chromatiaceae found in all cultures. Camp et al. studied the impact of a thermal stress event on the bacterial community composition of four Symbiodiniaceae cultures, finding again a high prevalence of Alphaproteobacteria, and particularly *Labrenzia* [41]. However, neither of these studies distinguished the planktonic bacteria from the bacteria closely associated with the algae. Furthermore, only extracellular bacteria have been unequivocally reported through scanning electron microscopy (SEM) [39, 42]. Actinobacteria and *Ralstonia* were reported to occur inside *in hospite* Symbiodiniaceae of the coral *Acropora granulosa*, using 2-dimensional confocal microscopy [43]. However, these observations remain unverified without 3-dimensional imaging to confirm whether such bacteria are truly inside the cell.

Here, we use a broad range of Symbiodiniaceae strains from six genera and unequivocally show the presence of intracellular and extracellular bacteria closely associated with both cultured and freshly isolated algae by 3-dimensional fluorescence in situ hybridization (FISH) confocal laser scanning microscopy (CLSM) and SEM. By combining culture-dependent and -independent techniques, we further characterize the composition of extracellular and intracellular bacteria in 11 Symbiodiniaceae cultures from nine different species. Our findings extend current knowledge of intracellular symbiosis, providing unique new insights into the biology of Symbiodiniaceae, and set the stage for the study of bacterial functions in Symbiodiniaceae, and more broadly in the cnidarian holobiont.

## Material and methods

Additional methods (CLSM, SEM, flow cytometry, and bacteria pure culturing) are available in the supplementary information.

### Symbiodiniaceae cultures

Symbiodiniaceae cultures (Table S1) were provided by the Australian Institute of Marine Science (AIMS, Townsville, QLD, Australia), except for *Breviolum minutum* which was isolated at the Marine Microbial Symbiont Facility (University of Melbourne, VIC, Australia) [44], and *Symbiodinium tridacnidorum* which was obtained from the Australian National Algae Culture Collection, CSIRO. Nine of the 11 strains were isolated from scleractinian corals, one from a clam (*Tridacna maxima*) and one from the anemone *Exaiptasia diaphana*. Symbiodiniaceae were grown in sterile 50 mL polypropylene (Falcon) culture flasks in 15 mL of sterile 1× IMK liquid media (Diago’s IMK Medium for marine microalgae, 1% w/v, NovaChem) prepared with filter sterilized Red Sea Salt water (fRSS). Growth of Symbiodiniaceae cultures was maintained by fortnightly transfers of cultures into fresh media and incubation at 26 °C on a 12:12 h light:dark cycle, with lighting at 50–70 μmol photons m^−2^ s^−1^ (Taiwan Hipoint Corporation, model 740FHC LED, light chambers).

### Symbiodiniaceae sampling and fixation for fluorescence in situ hybridization (FISH)

Symbiodiniaceae cells were sampled and twice washed by centrifugation at 5000 × *g* for 5 min, supernatant removed, and resuspended in phosphate buffered saline (PBS, pH 7.4) before being fixed in ice-cold 66% ethanol for 2 h at 4 °C. To minimize Symbiodiniaceae autofluorescence during downstream observations, cells were then photobleached by placing the samples under bright white light (LED) of ~400 µmol photons m^−2^ s^−1^ for at least 2 h at ambient temperature. Cells were washed in PBS and stored in 70% ethanol at −20 °C.

### Freshly isolated Symbiodiniaceae from *Exaiptasia diaphana* and *Galaxea fascicularis*

Five *E. diaphana* polyps [45] were placed in a glass tank and submerged in a solution of 0.39 M magnesium chloride in PBS for 30 min to anesthetize them. Tentacles were then removed with scissors and placed in 1.5 mL microcentrifuge tubes in 200 μL fRSS. The tissue was homogenized with a sterile micro-pestle.

*G. fascicularis* colonies were purchased from Cairns Marine in February 2020. These colonies were collected mid-February on Sudbury Reef in the Great Barrier Reef (GBR). They were kept in the Cairns Marine aquaria for 2 weeks and subsequently shipped to the University of Melbourne. In the University of Melbourne laboratory, corals were kept in RSS (34 ppt salinity) at 25.5 °C, on a 10:14 h light:dark cycle with lighting at 160–180 μmol photons m^−2^ s^−1^. In July 2020, four *G. fascicularis* polyps were detached from a single colony. To remove tissues from the skeleton, polyps were sprayed with pressured seawater (sterilized through a 0.22-µm filter) using a water flosser (Waterpik) with a Pik Pocket tip. Tissues were centrifuged at 825 × *g* for 5 min and homogenized with a sterile micro-pestle.

Freshly isolated Symbiodiniaceae cells were thrice washed by centrifugation at 825 × *g* for 5 min and resuspension in fRSS. After the last wash, pelleted cells were fixed in ice-cold 66% ethanol. Cells were then photobleached by placing the samples under bright white light (LED) of ~400 µmol photons m^−2^ s^−1^ for at least 2 h at ambient temperature. Cells were washed in PBS and stored in 70% ethanol at −20 °C.

The *E. diaphana* genotype used in this study [45] was previously shown to harbor *B. minutum* [44], while corals of the *Galaxea* genus from the GBR usually harbor Symbiodiniaceae of the *Cladocopium* and *Durusdinium* genera [46, 47].

### FISH

Teflon-printed microscope slides (ProSciTech) were coated with poly-L-lysine solution (0.01%) in PBS by aliquoting the solution onto printed wells, incubated at 37 °C for 3 h, then thrice washed in sterile Milli-Q water and air dried. Aliquots of 5–10 μL were pipetted into a well on treated ten-well slides. FISH was performed as previously described [48]. Samples on glass slides were air dried and permeabilized for 10 min in Triton X-100 solution (0.1% v/v) in PBS, and dehydrated in an ethanol series (3 min each in 50%, 80%, and 98% ethanol). Hybridization was in 18 μL buffer (0.9 M NaCl, 20 mM Tris-HCL pH 7.2, formamide [varied to accommodate optimal stringency for different probes—Table S2], 0.01% SDS) and 2 μL 16S rRNA targeting oligonucleotide probes at a final concentration of 5 ng/μL for 2 h at 46 °C. All probe sequences are listed in Table S2 [49–52]. When two different probes were used on the same sample, both probes had a final concentration of 5 ng/μL. Samples were then washed in a prewarmed buffer (NaCl [varied to accommodate optimal stringency for different probes—Table S2], 20 mM Tris-HCL pH 7.2, 0.01% SDS) for 15 min at 48 °C, briefly washed in ice-cold water and carefully dried. Slides were mounted with CitiFluor™ CFM3 mounting medium (Hatfield, PA, USA) and stored in the dark at −20 °C until observation in confocal microscopy.

### Symbiodiniaceae sampling for 16S rRNA gene metabarcoding

**Fig. 1:**
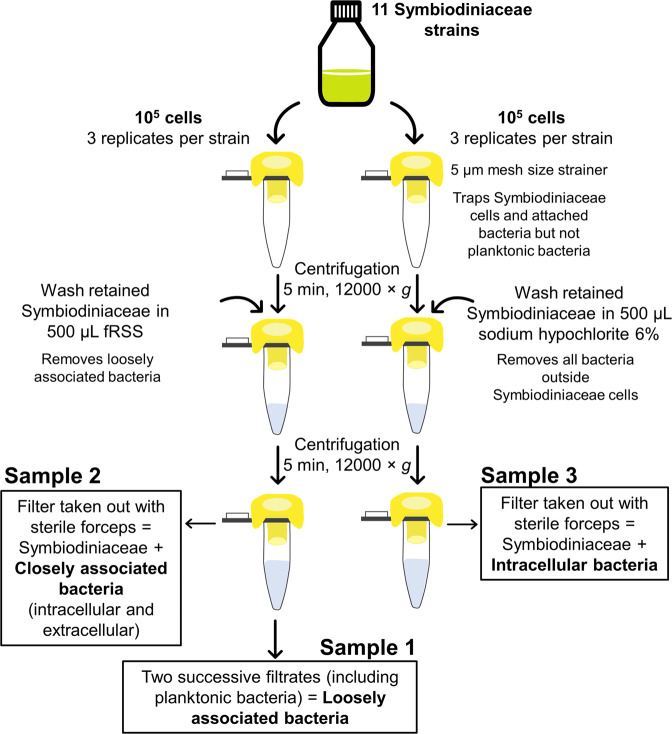
Experimental design for Symbiodiniaceae sampling for 16S rRNA gene metabarcoding. Aliquots of 10^5^ Symbiodiniaceae cells were transferred to 5 µm mesh size strainers and centrifuged to remove planktonic bacteria. To remove loosely associated bacteria (left), Symbiodiniaceae cells stuck on the strainer were washed with fRSS. Both successive filtrates, including planktonic bacteria, are referred to as ‘loosely associated bacteria’. Filters were recovered with sterile forceps, having kept Symbiodiniaceae cells and bacteria attached to the Symbiodiniaceae cell exterior and intracellular (closely associated). Another aliquot (right) was washed with sodium hypochlorite 6% to kill and remove extracellular bacteria, so that only intracellular bacteria remained in the sample. Three filters onto which no Symbiodiniaceae cells were deposited were sampled as negative controls.

The experimental design for this part of the study is outlined in Fig. 1. Sampling was performed between 2 and 4 p.m. (8–10 h into the light cycle) on the same day for every strain, during the exponential growth phase. For each culture used (Table S1), a total of six samples of 10^5^ cells were collected and filtered through individual 5 µm mesh strainers (pluriSelect, Germany). Strainers were sealed and centrifuged for 5 min at 12,000 × *g* to separate planktonic bacteria (<5 µm) from Symbiodiniaceae cells (>5 µm). For three of those samples, loosely attached bacteria were separated from Symbiodiniaceae cells by pipetting 500 µL fRSS onto the Symbiodiniaceae cells collected in the strainer, followed by centrifugation for 5 min at 12,000 × *g*. Filtrates (initial culture medium containing planktonic bacteria + fRSS wash) were snap frozen and represent ‘loosely associated bacteria’ **(**Fig. 1, Sample 1). Filters, having retained algal cells, intracellular bacteria and bacteria tightly attached to the algal cell’s exterior, were extracted from the strainer with sterile forceps, put into a sterile 1.5 mL microcentrifuge tube, and snap frozen in liquid nitrogen, and represent ‘closely associated bacteria’ **(**Fig. 1, Sample 2). For each culture used, the other three samples of 10^5^ cells were collected, filtered through a 5 µm mesh strainer (pluriSelect, Germany), sealed, and centrifuged for 5 min at 12,000 × *g*. To kill and remove all extracellular bacteria, retained Symbiodiniaceae cells were washed by pipetting 500 µL 6% sodium hypochlorite (bleach) onto the strainers, which were then sealed and centrifuged for 5 min at 12,000 × *g*. Filtrates containing bleach were discarded. Filters, having retained Symbiodiniaceae cells and only intracellular bacteria, were removed from the strainer with sterile forceps, put into a sterile 1.5 mL microcentrifuge tube, and snap frozen (Fig. 1, Sample 3). Hence, each culture yielded nine samples: three ‘loosely associated bacteria’ true replicates, three ‘closely associated bacteria’ true replicates, and three ‘intracellular bacteria’ true replicates. Three clean filters (onto which no algae cells were added) were extracted and snap frozen, as negative controls.

### DNA extraction for 16S rRNA gene metabarcoding

DNA extractions were performed using a salting-out method [53] with modifications as previously described [54]. Two blank DNA extractions were conducted as negative controls. A mock community, ZymoBIOMICS Microbial Community DNA Standard (Zymo Research), was included to check sequencing and processing quality.

### 16S rRNA gene PCR amplification, library preparation, and sequencing

Hypervariable regions V5–V6 of the 16S rRNA genes were amplified using the primer set 784F (5ʹ GTGACCTATGAACTCAGGAGTCAGGATTAGATACCCTGGTA 3ʹ) and 1061R (5ʹ CTGAGACTTGCACATCGCAGCCRRCACGAGCTGACGAC 3ʹ) [55]. Illumina adapters were attached to the primers and are shown as underlined. Bacterial 16S rRNA genes were PCR-amplified on a SimpliAmp Thermal Cycler (Applied Biosystems, ThermoFisher Scientific). Each reaction contained 1 μL of DNA template, 1.5 μL of forward primer (10 μM stock), 1.5 μL of reverse primer (10 μM stock), 7.5 μL of MyTaq HSRed MasterMix (BioLine), and 3.5 μL of nuclease-free water (Thermofisher), with a total volume of 15 μL per reaction. Three triplicate PCRs were conducted for each sample and three no template PCRs were conducted as negative controls. PCR conditions for the 16S rRNA genes were as follows: initial denaturation at 95 °C for 3 min, then 18 cycles of: denaturation at 95 °C for 15 s, annealing at 55 °C for 30 s, and extension at 72 °C for 30 s; with a final extension at 72 °C for 7 min. Samples were then held at 4 °C. Following PCR, triplicates were pooled, resulting in 45 μL per sample.

Metabarcoding library preparation and sequencing was performed at the Walter and Eliza Hall Institute (WEHI) in Melbourne, Victoria on one MiSeq V3 system (Illumina) with 2 × 300 bp paired-end reads. Library preparation involved addition of 20 μL of next-generation sequencing magnetic beads to 20 μL of PCR product (1:1), for clean-up to ensure high quality downstream sequencing. Beads were washed twice with 70% ethanol, and DNA was resuspended with 40 μL of nuclease-free water. Ten microliters of cleaned-up PCR products was combined with 10 μL 2× Taq MasterMix (M0270L, New England BioLabs) and 0.25 μM of forward and reverse indexing primers. The second PCR conditions were as follows: initial denaturation at 95 °C for 3 min, 24 cycles of: 9 °C for 15 s, 60 °C for 30 s, and 72 °C for 30 s; followed by a final extension at 72 °C for 7 min. Product size and specificity of two replicates of representative 16S rRNA gene amplifications were assessed using a TapeStation (2200 TapeStation, Agilent Technologies). A final bead clean-up (0.8 bead/PCR product ratio) was performed on a pool of 5 μL from each well per plate. Pooled libraries were checked for quality control, size determination, quantity, and purity of each sample, to inform pool normalization by using the TapeStation (2200 TapeStation, Agilent Technologies).

### Bacterial 16S rRNA gene analysis

QIIME2 v 2019.4.0 [56] was used for processing 16S rRNA gene sequences. The plugin demux [56] was used to create an interactive plot to visualize the data and assess the quality, for demultiplexing and quality filtering of raw sequences. The plugin cutadapt [57] was used to remove the primers and MiSeq adapters. Plugin DADA2 [58] was used for denoising and chimera checking, trimming, dereplication, generation of a feature table, joining of paired-end reads, and correcting sequencing errors and removing low quality reads (Q-score < 30). Summary statistics were obtained using the feature table to ensure processing was successful. Taxonomy was assigned by training a naive Bayes classifier with the feature-classifier plugin [56], based on a 99% similarity to the V5–V6 region of the 16S rRNA gene in the SILVA 132 database to match the 784F/1061R primer pair used [59]. Alignment [60] and phylogeny [61] packages enabled the production of a phylogenetic tree for later analyses in R Studio. Mitochondria and chloroplast reads were filtered out. Metadata file, phylogenetic tree, and tables with Amplicon Sequence Variant (ASV) taxonomic classifications and counts were imported into R for statistical analyses.

### Statistical analyses in R studio

Statistical analyses and graphs were performed using R version 3.5.0 [62], and the packages phyloseq [63], vegan [64], RVAideMemoire [65], ggplot2 [66], tidyverse [67], indicspecies [68]. Statistical tests were considered significant at *α* = 0.05, unless otherwise stated. Metadata file, taxonomy table, phylogenetic tree, and ASV table were imported into R and mitochondria and chloroplast sequences were removed. Contaminant ASVs, arising from kit reagents and sample manipulation, were identified manually based on their abundance in negative controls: any ASV that was five times more abundant in the mean abundance of either filter blanks, extraction blanks or no template PCRs compared to the mean of all Symbiodiniaceae samples, and that represented at least 1000 reads in all Symbiodiniaceae samples, was considered a contaminant and removed from the dataset. Known contaminants (e.g., *Cutibacterium*) were also removed manually. Thirty putative contaminant ASVs were identified and removed, constituting 3.7% relative abundance of the bacterial communities in Symbiodiniaceae samples (Table S3). The mock community sample results validated the sequencing run.

Alpha-diversity metrics (observed ASVs, Simpson index, Shannon index) were calculated after rarefying the samples to 4800 reads per sample (the lowest read number for a given sample), a depth that was sufficient to capture diversity across samples, as the number of ASVs had reached an asymptote at this read number (Fig. S1). Alpha-diversity data were then analyzed for overall differences using the non-parametric Kruskal–Wallis test. Differences in community composition (β-diversity) were computed using Bray–Curtis dissimilarity matrices and tested via permutational multivariate analysis of variance (PERMANOVA). Variation in community composition among samples was visualized with PCoA. A test for multivariate homogeneity of group dispersions (PERMDISP) was used to check for homogeneity of variances and pairwise comparisons were performed between groups. Core genera were identified as genera that were present in all Symbiodiniaceae strains within one location. The indicator value analysis [68] was applied to detect genera that were significantly associated with a specific treatment when both specificity and fidelity had probabilities >75%.

## Results

### Intra- and extracellular bacteria of diverse taxonomic affiliations associate with cultured Symbiodiniaceae

**Fig. 2:**
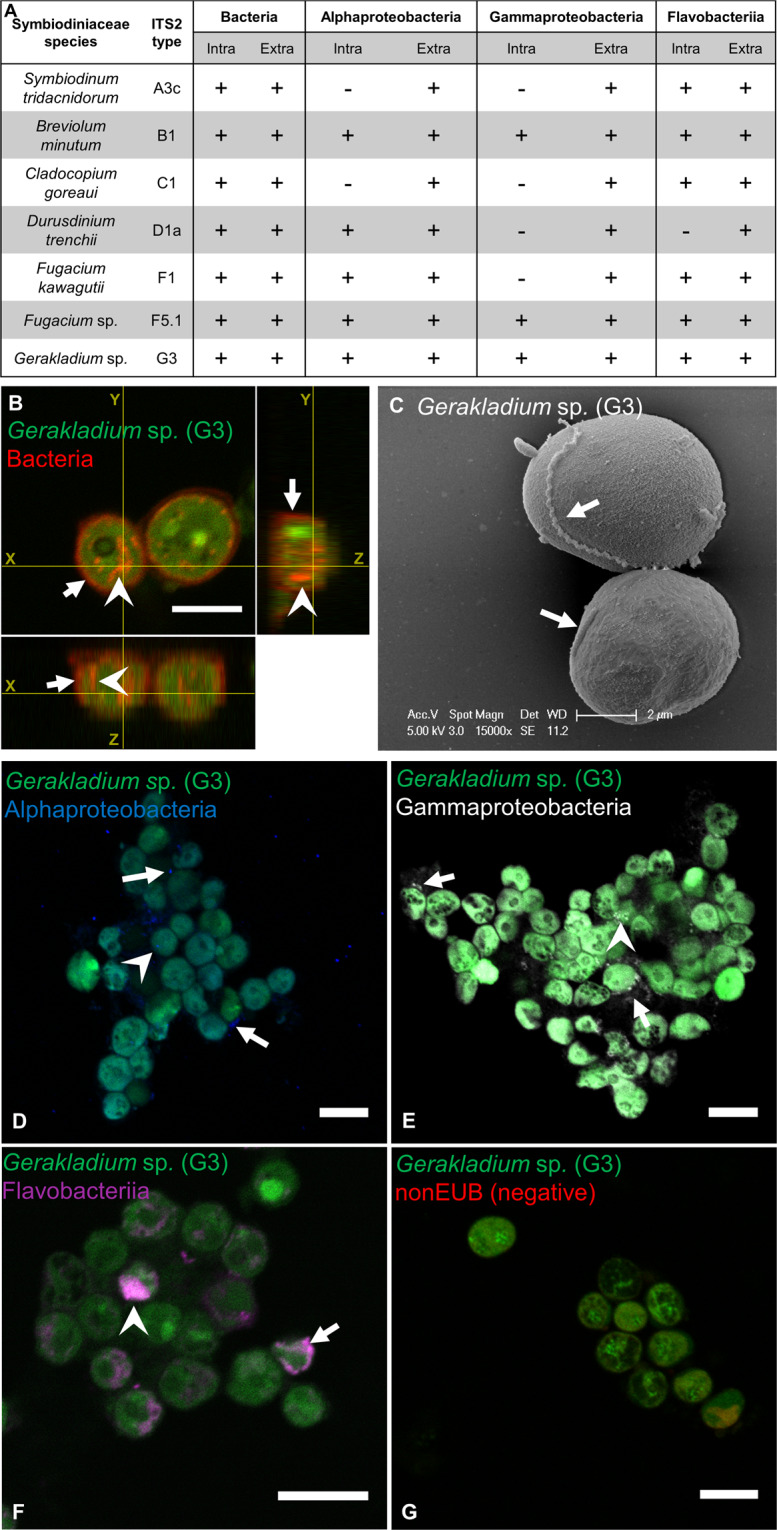
Symbiodiniaceae associate with intra- and extracellular bacteria of diverse taxonomic origin. **A** Summary table of bacterial occurrence in seven Symbiodiniaceae species based on FISH observations. Intra: intracellular; Extra: extracellular; ± signifies present/absent. **B** Orthogonal projections of a Z-stack of two *Gerakladium* sp. (G3) cells stained with the EUBmix probe observed in CLSM, highlighting the presence of both intra- and extracellular bacterial associates. **C** SEM photo of two *Gerkladium* sp. (G3) cells, highlighting the presence of bacteria attached to the cell exterior. Localization of FISH-stained Alphaproteobacteria (**D**), Gammaproteobacteria (**E**), and Flavobacteriia (**F**) in *Gerkladium* sp. (G3), observed in CLSM. Scale bar in **B**, **D**, **E**, **F** is 10 µm. Arrows point at extracellular bacteria and arrowheads point at intracellular bacteria. Green: Symbiodiniaceae; red: EUB338-mix probe (all bacteria); blue: Alf1B probe (Alphaproteobacteria); white: Gam42a probe (Gammaproteobacteria); magenta: CF319 probe (Flavobacteriia). **G** FISH negative control using the nonEUB probe. All scale bars are 10 µm (color figure online).

To characterize the bacterial communities associated with Symbiodiniaceae, we first used FISH and CSLM to localize bacteria in seven different Symbiodiniaceae cultures (Table S1). The use of the universal bacterial probes (EUB338-mix) revealed the presence of bacteria closely associated with cells of all Symbiodiniaceae species examined (Fig. 2A and S2). Orthogonal projections of Z-stacks showed that bacteria were localized both intra- and extracellularly (Fig. 2B). Extracellular bacteria occurred both close to the cell surface, and free in the culture (Figure S2). SEM confirmed the occurrence of extracellular bacteria attached to the algal surface in six tested cultures (Fig. 2C and S3). Dividing extracellular bacteria were observed (Fig. S3A), suggesting they are active and viable, and some bacteria occurred within extracellular polymeric substances (Fig. S3D), potentially indicating very close physiological association, despite still being extracellular.

Furthermore, FISH on the Symbiodiniaceae cultures using class-specific bacterial probes (Table S2) revealed that all Symbiodiniaceae species examined were associated with extracellular bacteria of Alphaproteobacteria, Gammaproteobacteria, and Flavobacteriia, but the taxonomic affiliation of the intracellular bacteria differed among Symbiodiniaceae species (Figs. 2A, D–F, and S2). Flavobacteriia were the most commonly occurring intracellular bacteria, with only *Durusdinium glynnii* not harboring this class. *B. minutum*, *Fugacium* sp. (F5.1) and *Gerakladium* sp. (G3) were the only Symbiodiniaceae cultures where intracellular bacteria hybridized with all probe types, indicating that divergent bacterial species co-occur intracellularly. Such co-occurrence was confirmed by combining class-specific probes: Gammaproteobacteria were found to co-occur intracellularly with both Alphaproteobacteria and Flavobacteriia in *B. minutum* (Fig. S4A, B), and with Flavobacteriia in *Gerakladium* sp. (G3) (Fig. S4C).

### Bacteria also associate with Symbiodiniaceae *in hospite*

**Fig. 3:**
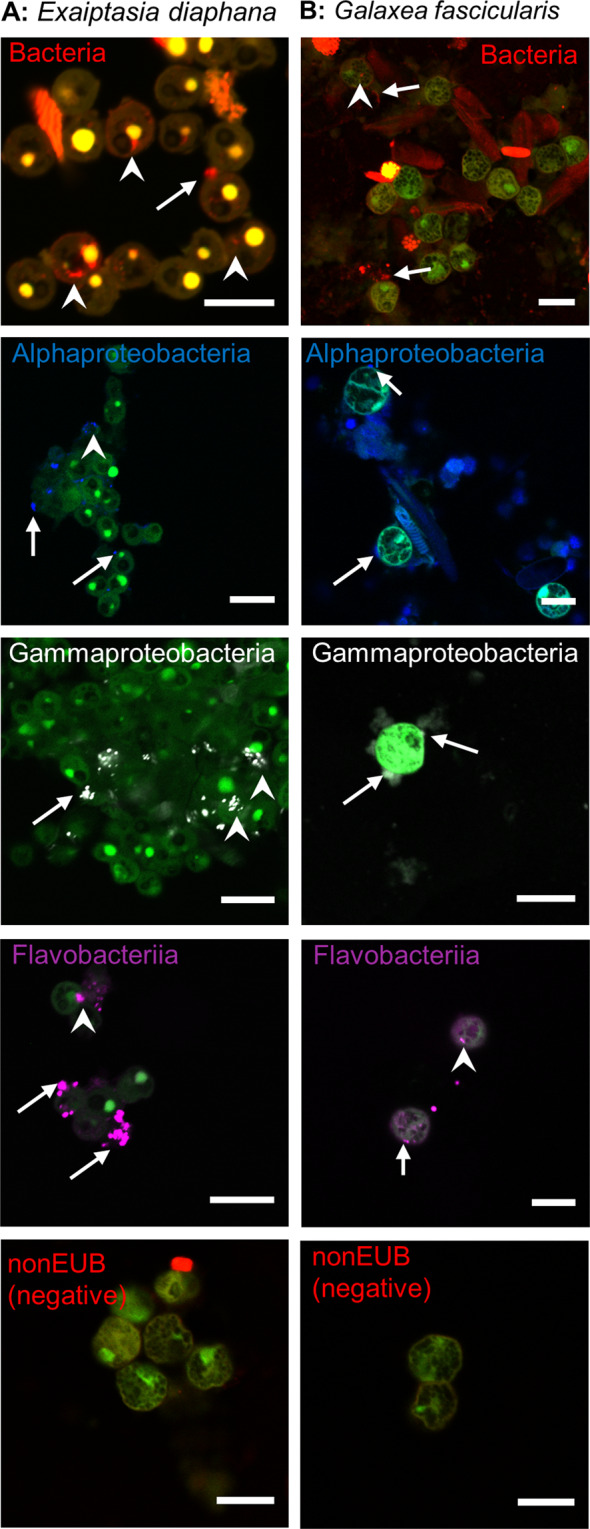
Bacteria associate with Symbiodiniaceae in cnidarian holobionts. Localization of FISH-stained bacteria in Symbiodiniaceae freshly isolated from the anemone *Exaiptasia diaphana* (**A**) and the coral *Galaxea fascicularis* (**B**), observed with CLSM. Scale bar is 10 µm in all photos. Arrows point at extracellular bacteria and arrowheads point at intracellular bacteria. Green: Symbiodiniaceae; red: EUB338-mix probe (all bacteria); blue: Alf1B probe (Alphaproteobacteria); white: Gam42a probe (Gammaproteobacteria); magenta: CF319 probe (Flavobacteriia) (color figure online).

To assess whether the observed bacteria–Symbiodiniaceae associations were not artefacts of extended laboratory culture, we freshly isolated Symbiodiniaceae from two cnidarians: the sea anemone *E. diaphana* and the scleractinian coral *G. fascicularis*. FISH probing and CSLM of freshly isolated Symbiodiniaceae from *E. diaphana* showed that Alphaproteobacteria, Gammaproteobacteria, and Flavobacteriia were all found both intra- and extracellularly (Fig. 3A). In *G. fascicularis*’ Symbiodiniaceae, only Flavobacteriia were found intracellularly, while all three bacterial classes were observed extracellularly (Fig. 3B). These findings indicate that *in hospite* Symbiodiniaceae also closely associate and interact with bacteria.

### Cell cycling and sampling time influences bacterial prevalence in *Cladocopium goreaui*

**Fig. 4:**
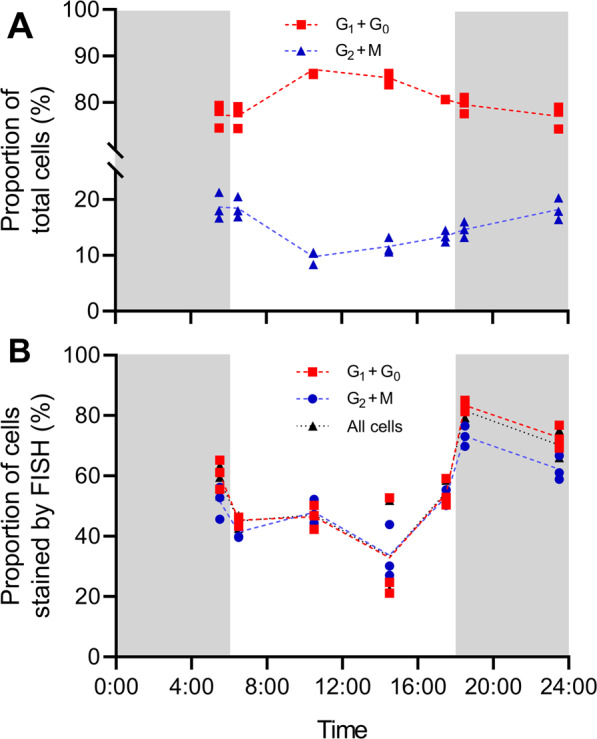
Sampling time and cell cycle phase influence Symbiodiniaceae-bacteria association in *Cladocopium goreaui*. **A** Proportion of cells either in G_1_ or G_0_ phase (red) and G_2_ or M phase (blue) across a day, as assessed by PI staining analyzed by flow cytometry. **B** Proportion of cells stained by FISH in either all cells (black), cells in G_1_ and G_0_ phase (red), or cells in G_2_ and M phase (blue), as analyzed by flow cytometry. For each time point, three independent replicates from three cultures are represented, with dotted lines linking the median of each condition. Gray-shaded areas represent dark time, while white areas represent light time (color figure online).

Bacterial abundances differed between cells within samples and between Symbiodiniaceae species. Symbiodiniaceae follow a day–night cell cycle with cell division happening at dawn [69]. We hypothesized that the different physiological, metabolic, and morphological cellular states might impact the abundance, location, and composition of associated bacteria. Thus, we sampled *C. goreaui* at regular intervals for 24 h, combined FISH with propidium iodide (PI) staining to compare bacterial prevalence in the G_1_ and G_2_ phases of the algal cell cycle, and processed samples by flow cytometry to quantify the proportion of Symbiodiniaceae associated with bacteria (Fig. S5). PI staining revealed a higher proportion of Symbiodiniaceae cells in G_2_ or M phase during the night (Fig. 4A), consistently with cell division occurring at dawn. Within a sample, not all cells were stained by FISH (Fig. 4B, dotted black lines), suggesting that not all cells are associated with bacteria at any given time. The proportion of stained cells varied between 33% at 2:30 p.m. and 82% at 6:30 p.m. immediately after the lights were turned off and was consistently higher at night. Time of sampling was a statistically significant factor in these variations (ANOVA; *F*_(6,14)_ = 16.1; *p* < 0.0001). When separating samples by their cell cycle phase as determined by the PI staining, the same trend was observed, with the proportion of stained cells being higher at night for both groups **(**Fig. 4B, red and blue lines). In addition, cells in G_1_ or G_0_ phase showed a higher proportion of stained cells than cells in G_2_ or M phase. Both sampling time and cell cycle phase were statistically significant factors, but their interaction was not (ANOVA; *F*_time(6,28)_ = 34.8, *p*_time_ < 0.0001; *F*_cell cycle(1,28)_ = 5.8, *p*_cell cycle_ = 0.02; *F*_interaction(6,28)_ = 1.1*, p*_interaction_ = 0.41). Thus, while it is evident that Symbiodiniaceae cells tend to more readily associate with bacteria during the night, the cell cycle phase does not explain this variability, as cells in G_1_ or G_0_ phase show a higher proportion of stained cells regardless of sampling time.

### Culture-dependent and -independent methods reveal diverse intracellular and extracellular bacterial communities

Pure culturing of bacteria associated with Symbiodiniaceae cultures revealed the existence of diverse bacterial communities. Pure bacterial isolates (*n* = 141) from six Symbiodiniaceae species were identified through phylogenetic analysis of their 16S rRNA gene sequences (Fig. S6, Dataset S1) and found to span 20 genera within four classes: Alpha- and Gammaproteobacteria, Flavobacteriia, and Bacilli. Only *Muricauda* (Flavobacteriia) was cultured from all Symbiodiniaceae species. Because the bacterial diversity revealed from pure-culturing methods likely under-represents the actual diversity, we also implemented a culture-independent method—16S rRNA gene metabarcoding. Each algal culture was fractionated through washes of different stringency in three sub-components, called loosely associated, closely associated (intracellular and tightly attached to the outer surface of Symbiodiniaceae cells), and intracellular bacteria (see Fig. 1 and material and methods for detailed experimental design). These three groups are hereafter referred to as the ‘location’ factor. SEM of *B. minutum* and *Fugacium* sp. (F5.1) cells confirmed that washing the cells with sodium hypochlorite removed any extracellular bacteria while keeping the Symbiodiniaceae cells intact, while washing them with fRSS removed some, but not all, cell surface-attached bacteria, suggesting that only closely attached bacteria remain (Fig. S7). Sequencing produced 7,747,433 reads across 11 Symbiodiniaceae cultures (*n* = 3 per culture per treatment; 3 treatments; 99 samples in total), filter controls (*n* = 3), DNA extraction blanks (*n* = 2), and no template PCRs (*n* = 4). Four samples were removed because they had low-read counts (<1000 reads). After merging, denoising and chimera filtering 4,831,092 reads remained. After removal of contaminants, 884 ASVs were observed across the remaining samples.

**Fig. 5:**
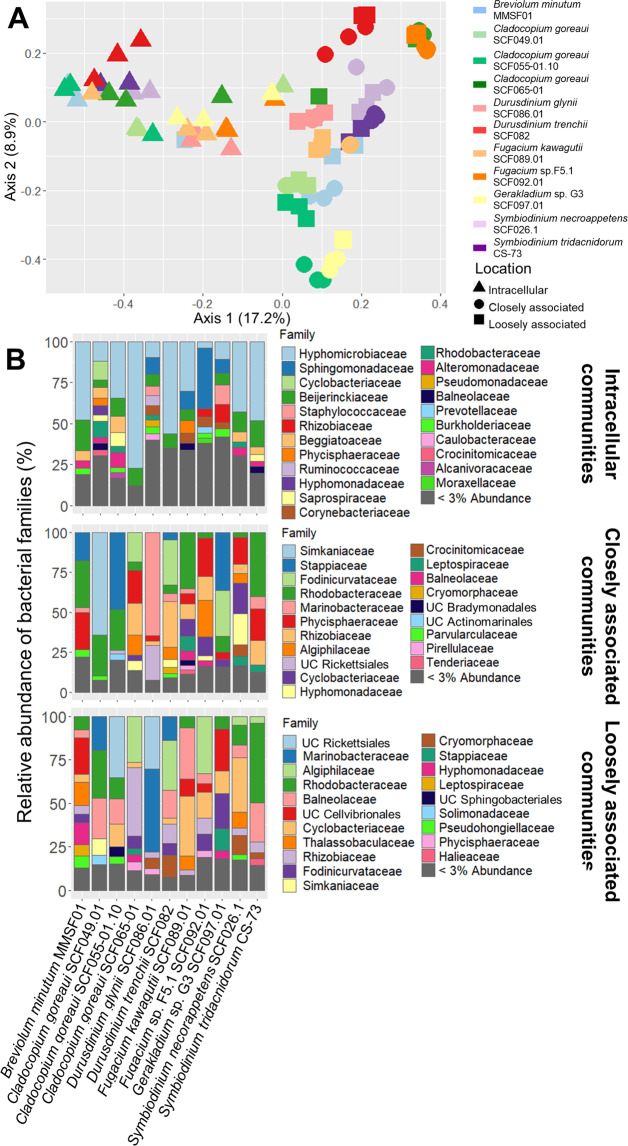
Bacterial community composition strongly varies based on location and Symbiodiniaceae strain. **A** PCoA visualization of beta-diversity of the bacterial communities in 11 Symbiodiniaceae strains and three locations, based on Bray–Curtis dissimilarity matrices. Each point is an individual sample. Both location and Symbiodiniaceae strain, as well as their interaction, had significant effects based on PERMANOVA testing. **B** Relative abundance of bacterial families in Symbiodiniaceae’s intracellular (upper panel), closely associated (middle panel), and loosely associated (lower panel) communities. For each strain × location combination, three independent replicates were merged. UC unclassified.

One out of three alpha-diversity metrics (observed ASVs) showed significant differences among Symbiodiniaceae locations (Kruskal–Wallis test; *χ*^2^_ObsASBs(2)_ = 8.7, *p* = 0.01) and Symbiodiniaceae strains (Kruskal–Wallis test; *χ*^2^_ObsASBs(10)_ = 37.3, *p* < 0.001), while Shannon’s index only showed differences among Symbiodiniaceae locations (Kruskal–Wallis test; *χ*^2^_Shan(2)_ = 8.8, *p* = 0.01) (Fig. S8A, B). The Simpson index did not show any difference among locations (Kruskal–Wallis test; *χ*^2^_Simp(2)_ = 2.5, *p* = 0.28) or Symbiodiniaceae strains (Kruskal–Wallis test; *χ*^2^_Simp(10)_ = 14.9, *p* = 0.13). Principal coordinate analysis (PCoA) visualization of β-diversity using the Bray–Curtis dissimilarity index revealed a clear overlap between closely and loosely associated communities, while intracellular communities formed a separate cluster (Fig. 5A). Statistical analyses showed that community structure varied with Symbiodiniaceae location and strain, and their interaction (PERMANOVA, Bray–Curtis method, 999 permutations; *F*_strain(10,62)_ = 11.5, *p* < 0.001; *F*_location(2,62)_ = 27.4, *p* < 0.001; *F*_interaction(20,62)_ = 5.3, *p* < 0.001). Pairwise PERMANOVA comparisons between locations confirmed all three were statistically different from each other (*p* = 0.001 for each pairwise comparison). However, the PCoA representation highlighted that, for a given strain, closely and loosely associated communities were often similar. PCoAs confirmed that intracellular communities from different Symbiodiniaceae strains overlap (Fig. S8C), while closely and loosely associated communities are clearly separated based on strain (Fig. S8D, E). Community composition at the family level highlighted those dissimilarities (Fig. 5B). Intracellular communities were dominated by Hyphomicrobiaceae, Beijerinckiaceae, and Sphingomonadaceae (Fig. 5B, upper panel); closely associated communities were dominated by Rhodobacteraceae, Marinobacteraceae, Phycisphaeraceae, Rhizobiaceae, and Stappiaceae (Fig. 5B, middle panel); loosely associated communities were dominated by Rhodobacteraceae, Marinobacteraceae, Algiphilaceae, and Balneolaceae (Fig. 5B, lower panel).

Genera that yielded culturable isolates from at least four of the six Symbiodiniaceae species (Fig. S6) were specifically investigated in the metabarcoding data. *Marinobacter, Labrenzia*, and *Roseitalea* were consistently abundant in most samples (Figure S9). *Muricauda* was present in most samples, but with a relative abundance below 1% in all samples (Figure S9). The prevalence and abundance of *Bacillus* and *Roseovarius* were very low, with *Bacillus* being present in only three Symbiodinaceae strains, contrasting with their high prevalence in cultured isolates. Intriguingly, typically intracellular taxa, such as Simkaniaceae and Rickettsiales, were found in high abundances both in closely and loosely associated communities, particularly in *C. goreaui* and *Durusdinium glynii*, but in low abundance in intracellular communities (Fig. 5B, Dataset S2). Simkaniaceae alternate between infectious extracellular elementary bodies, and intracellular replicative reticulate bodies [70, 71]. Hence, high numbers of Simkaniaceae elementary bodies might have been released, through vesicles or host cell lysis, at the time of sampling, explaining their high abundance in extracellular communities.

### Investigation of core bacterial communities in Symbiodiniaceae

**Fig. 6:**
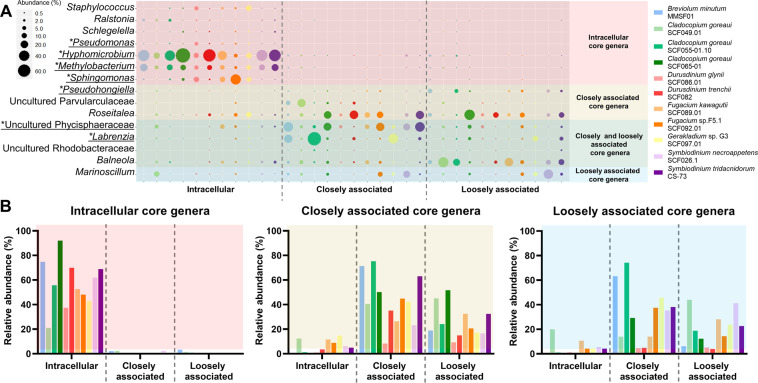
Core genera are specific in intracellular communities, less so in closely and loosely associated communities. **A** Relative abundance of genera found in every Symbiodiniaceae strains in either intracellular, closely associated, or loosely associated samples—‘core genera’. For each value, three independent replicates were merged. Asterisk in front of an underlined genus signifies that the genus was also detected through an indicator genera analysis. **B** Relative abundance of the sum of all core genera from intracellular (left panel; seven genera), closely associated (middle panel; seven genera), or loosely associated (right panel; five genera) communities.

A large number of genera (325) were present among the 884 ASVs observed across all samples. Seven genera were found in intracellular communities of all 11 Symbiodiniaceae strains, seven in closely associated communities of all strains, and five in loosely associated communities of all strains (Fig. 6A). These genera are hereafter referred to as ‘core genera’ for their respective location. No genus was found to occur in all Symbiodiniaceae strains and all locations. Intracellular core genera accounted for an average of 57% of all reads in intracellular communities across all strains (Fig. 6B, left panel, Table S4, and Dataset S3A), with *Hyphomicrobium*, *Methylobacterium,* and *Sphingomonas* being the most abundant. Intracellular core genera had very low abundance in closely and loosely associated communities (<1% of all reads for both locations in all strains) (Fig. 6B, left panel, Table S4, and Dataset S3A), suggesting they are specifically present in intracellular communities. Conversely, closely associated core genera accounted for 44%, 26%, and 6% in closely associated, loosely associated and intracellular communities, respectively (Fig. 6B, middle panel, Table S4, and Dataset S3B); likewise, loosely associated core genera accounted for 33%, 20%, and 5% in closely associated, loosely associated and intracellular communities, respectively (Fig. 6B, right panel, Table S4, and Dataset S3C). This suggests that closely and loosely associated communities show a degree of similarity that they do not share with intracellular communities, which is consistent with the PCoA results. Finally, an indicator value analysis was performed to identify genera that represent indicators for each location. Eight genera were identified: *Pseudomonas, Hyphomicrobium, Methylobacterium*, and *Sphingomonas* for intracellular communities; *Labrenzia*, uncultured Phycisphaeraceae, and uncultured Kiloniellaceae for closely associated communities; *Pseudohongiella* for loosely associated communities. All but uncultured Kiloniellaceae were already present in the core genera analysis and are underlined in Fig. 6A. Uncultured Kiloniellaceae had an abundance lower than 1% in all but two samples (*C. goreaui* SCF049.01—closely associated; *Fugacium kawagutii*—Closely associated).

## Discussion

Despite coral-Symbiodiniaceae interactions having been extensively studied, and coral–bacteria interactions recently gaining traction, Symbiodiniaceae–bacteria interactions remain understudied. Deciphering these interactions in *ex hospite* Symbiodiniaceae is the first step toward understanding Symbiodiniaceae–bacteria cooperation within the coral holobiont. Here, we describe the bacterial microbiome of a wide diversity of cultured Symbiodiniaceae. Notably, through three-dimensional CLSM and SEM on long-term cultures of six genera, we provide unequivocal evidence that bacteria are present inside Symbiodiniaceae cells, as well as on the cell’s exterior. We show the occurrence of three different classes within, and outside, Symbiodiniaceae cells: Alpha- and Gammaproteobacteria, and Flavobacteriia. In addition, different classes were found to co-occur within the same Symbiodiniaceae cell, suggesting the possibility of inter-class, intracellular cooperation in this symbiosis. We also show the presence of intra- and extracellular bacteria in Symbiodiniaceae freshly isolated from two cnidarians, suggesting that Symbiodiniaceae–bacteria interactions might be similar within cnidarian hosts. Additional work will be needed on *in hospite* Symbiodiniaceae to confirm that our results obtained in long-term cultures are generalizable to cnidarian holobionts.

Metabarcoding data indicated that bacterial communities associated with Symbiodiniaceae cultures were largely comprised of *Hyphomicrobium*, *Methylobacterium*, *Sphingomonas*, *Marinobacter*, *Labrenzia*, *Balneola*, *Algiphilus*, Rhodobacteraceae, Phycisphaeraeae, and *Roseitalea*. *Marinobacter, Labrenzia*, and *Muricauda* were previously found to be abundant in a wide range of Symbiodiniaceae cultures [39–41], indicating the conservation of specific bacterial associates across Symbiodiniaceae species, coral hosts, and coral host location. *Marinobacter* enhances the growth of *Scrippsiella trochoidea*, presumably by producing vibrioferrin, a siderophore that promotes iron uptake by the dinoflagellate [18]. *Labrenzia* has the ability to produce and degrade DMSP [72, 73], a compound that has ROS-scavenging abilities [74], and thus could have functions in overall ROS regulation, or excess ROS scavenging during coral bleaching events. *Muricauda* produces zeaxanthin, a carotenoid with ROS-scavenging abilities, and is known to increase heat and light resistance in cultured Symbiodiniaceae [19]. Through these functions, it is thus likely that *Marinobacter, Labrenzia*, and *Muricauda* are beneficial symbionts of Symbiodiniaceae.

Unlike previous studies, our metabarcoding experiment distinguished between intracellular, closely associated, and loosely associated bacterial communities. Closely and loosely associated communities were similar to each other in any given Symbiodiniaceae strain, while intracellular communities were different from those in both extracellular locations. Furthermore, intracellular communities showed high similarity among strains, as evidenced by the fact that the seven intracellular core genera accounted on average for more than 50% of all intracellular reads. Interestingly, core genera from closely and loosely associated communities largely overlapped within a given Symbiodiniaceae strain. This indicates that the closely associated communities were dominated by extracellular bacteria that were also present in the loosely associated communities. It is possible that loosely associated bacteria are derived from closely associated bacteria and can live freely in the algal culture medium, reinforcing the importance of the phycosphere in shaping extracellular bacterial assemblages. Whether the phycosphere promotes the establishment of intracellular bacteria remains unclear, and the factors attracting bacteria and allowing them to enter Symbiodiniaceae cells, in culture but also *in hospite*, need to be addressed. While extracellular associates could come from extracellular bacteria naturally associating with Symbiodiniaceae in cnidarian hosts or in seawater, they also might have been accidentally introduced, and subsequently maintained, during the culturing process. Comparing bacterial communities between cultured and *in hospite* Symbiodiniaceae will shed light on this question.

The core genera analysis allowed us to identify conserved bacterial genera across Symbiodiniaceae strains, which might support crucial functions for the Symbiodiniaceae. Among intracellular core genera, *Hyphomicrobium, Methylobacterium*, and *Sphingomonas* were the most abundant. *Hyphomicrobium* can metabolize dimethylsulfate [75], and could thus be involved in sulfur cycling. Incidentally, this process is improved when co-cultured with *Pseudomonas* [75], another intracellular core genus. *Hyphomicrobium* species can denitrify using methanol as a carbon source [76]. Interestingly, *Methylobacterium* is methylotrophic, and can also use methanol, and other short carbon compounds, as a carbon source. Whether there is sufficient methanol production within Symbiodiniaceae cells to support such activity is unknown. *Sphingomonas* has previously been found intracellularly in the toxic dinoflagellate *Alexandrium minutum* [77]. Some *Sphingomonas* species are able to fix nitrogen in rice plants [78], and hence could be involved in nitrogen cycling within Symbiodiniaceae cells. Interestingly, *Ralstonia*, a core intracellular genus here, was found to closely associate with *in hospite* Symbiodiniaceae in the coral *A. granulosa* [43], although its role remains elusive.

Unexpectedly, intracellular communities showed the highest richness. Nonetheless, community composition analysis indicates that some cultures are dominated by very few families: only two families show relative abundance above 3% in the intracellular communities of *C. goreaui* SCF055.01-10 and *D. trenchii* SCF082, and these two families account for more than 60% of the reads. Low-abundance bacteria detected in intracellular communities might therefore not be present in all the cells in the sample and could be the result of heterotrophy. Indeed, Symbiodiniaceae have been shown to engulf and feed on bacteria [79]. While it is possible that low-abundance families are the result of unspecific heterotrophic feeding, several lines of evidence point at a significant proportion of bacteria being true intracellular symbionts: (1) intracellular bacteria were detected in Symbiodiniaceae freshly isolated from cnidarian hosts, where a bacterium encounter and engulfment is less likely than in culture; (2) flow cytometry and FISH showed a high proportion of cells associated with intracellular bacteria, and sometimes had high abundances of intracellular bacteria, during the day when heterotrophy is minimal (Jeong et al. [79] show a maximum of 6 *Synechococcus* cells being ingested per Symbiodiniaceae cell per hour); (3) we show the existence of a specific and conserved intracellular community that is low in abundance in extracellular communities.

Finally, we provide evidence that the Symbiodiniaceae microbiome is dynamic. We demonstrate that daily cycling and sampling time, especially light, influence the proportion of Symbiodiniaceae cells that are closely associated with bacteria. This is concordant with previous studies that showed Symbiodiniaceae bacterial communities change with temperature [41], and that bacterial density increases with culture age in the dinoflagellates *Heterocapsa circularisquama, Alexandrium catenella*, and *Protoceratium reticulatum* [11, 80]. It was also shown in *H. circularisquama* that the density of intracellular bacteria increases when the dinoflagellate culture is switched to continuous darkness [80], an increase we also report here during the night period. Several factors could explain such an increase when these dinoflagellates do not have access to light and thus cannot photosynthesize: (1) dark mitochondrial respiration modifies oxygen levels, pH and overall metabolite profiles within the cell as well as within the phycosphere, factors that could impact bacterial density and diversity; and (2) heterotrophy [79], which is higher in the dark [81].

In conclusion, the data presented in this study provide important new insights into Symbiodiniaceae–bacteria interactions in long-term cultures and to some extent *in hospite*. Future studies should focus on identifying the *in hospite* Symbiodiniaceae-associated bacterial communities in greater detail and assess whether Symbiodiniaceae-bacteria communities are similar *in* and *ex hospite*. Importantly, we show the existence of a complex intracellular consortium of bacteria, which is highly conserved across six cultured Symbiodiniaceae genera. Determining the function of these endosymbionts, and whether they might contribute to coral health, and potentially to bleaching resilience, will be key in fully understanding Symbiodiniaceae–bacteria interactions. Bacterial probiotics are increasingly brought forward as a potential solution to mitigate coral bleaching [33, 82–84]. ROS-scavenging bacteria able to intimately associate with Symbiodiniaceae may be strong candidates for probiotics as excessive levels of ROS, the putative cause of coral bleaching, originate within Symbiodiniaceae cells. Further, intracellular bacteria could be considered as targets for genetic modification, because their intracellular lifestyle might prevent them from spreading to the environment and other non-targeted organisms.

## Supplementary information

Supplementary information

Dataset S1

Dataset S2

Dataset S3

Table S3

Table S4

## Data Availability

Genbank accession numbers for 16S rRNA sequences of cultured bacteria are MT840521-MT840661 (see Dataset S1). Raw MiSeq data are available under NCBI BioProject ID PRJNA650221.
