# Supplementary material for: Intracellular bacteria are common and taxonomically diverse in cultured and in hospite algal endosymbionts of coral reefs
Source: ISME J. 2021 Feb 8;15(7):2028–42. doi: 10.1038/s41396-021-00902-4 (PMC8245515; doi:10.1038/s41396-021-00902-4)
Supplement: Supplementary file 1 — Supplementary information [file 41396_2021_902_MOESM1_ESM.docx]

**Supplementary methods**

**Confocal laser scanning microscopy (CLSM)**

Observations were made on either a Nikon AIR or a Nikon C2 confocal laser scanning microscope (Nikon, Tokyo, Japan) with the NIS325 Element software. Virtual band mode was used to acquire variable emission bandwidth to tailor acquisition for specific fluorophores. An oil immersion objective of 60X magnification and 1.4 numerical aperture was used for image acquisition. The fluorophores Cy3 and Atto550 were excited using the 561 µm laser line, Atto647 using the 640 µm laser line, and the Symbiodiniaceae autofluorescence using the 409 µm laser line with a detection range of 560-640 for Cy3, 540-590 nm for Atto550, 655-710 for Atto647 and 670-720 for Symbiodiniaceae. For 3D reconstructions of the Z-stacks, sections were acquired using Z steps of 0.2 μM. Nd2 files were processed using ImageJ. In Figure 2B, a whole Z-stack is shown. For more clarity, only single photos extracted for Z-stacks are presented in Figures 2D-G, 3, S2, and S4. Each photo was selected in the center of the Z-stack and covers 0.2 µm. As Symbiodiniaceae cells have a diameter of around 8-10 µm, FISH signal present within a Symbiodiniaceae cell represents intracellular bacteria.

**Scanning electron microscopy (SEM)**

A total of 10^6^ cells were collected from Symbiodiniaceae cultures **(Table S1)** and passed through 5 μm mesh size strainers (pluriSelect, Germany), washed with fRSS (for Figures 1 and S2) or the stipulated washing solution (for Figure S5), and centrifuged at 12,000 × *g* for 5 min. The retentate was resuspended in 200 μL IMK medium. A volume of 5 μL glutaraldehyde was added (2.5% final concentration), and cells were fixed for 1 h. Cells were then washed once in fRSS, once in fRSS/PBS (1:1 ratio), once in PBS and twice in milliQ water, and resuspended in 500 μL milliQ water. Coverslips (18 mm x 18 mm) were coated with poly-L-lysine solution (0.01%) as described above. Volumes of 100 μL of fixed cells were added onto the slide and cells were left to attach for 15 min at ambient temperature. The solution was then removed, and coverslips were gradually dehydrated in an ethanol series: 30 min each in 10%, 20%, 40%, 60%, 80%, 3 times 100%. Samples were kept overnight at 4°C in 100% ethanol. Critical point drying was performed in a CPD300 critical point dryer (Leica). Samples were coated with gold with a Quorum Q150T ES Plus for 2 min and observed with a XL30 scanning electron microscope (Philips), using a 5 kV beam.

**Sample preparation for flow cytometry**

Three independent flasks of a *C. goreaui* culture were used as three replicates. Seven time points per day (12 hr daylight, with lights on at 6:00 and off at 18:00 local time) were measured, with four day time-points (6:30, 10:30, 14:30, 17:30) and three night time-points (5:30, 18:30, 23:30). Approximately 3 ×10^6^ cells from each flask were sampled for each time point, fixed, photobleached, and stored as described above. Each sample was separated into four sub-samples: no staining, FISH with EUBmix-Atto647, PI staining, and both FISH with EUBmix-Atto647 and PI staining. FISH was performed as described above directly in the tubes: after each treatment, samples were centrifuged for 5 min at 3,000 × *g*, the solution was discarded, and the cells were resuspended in the next solution (500 µL of solution for each treatment). Hybridization was performed in 200 µL of hybridization buffer with either no probe (no staining, or PI staining only samples) or the EUBmix-Atto647 probe at a 5 ng/µL concentration. At the end of the FISH protocol, samples were washed with water, centrifuged for 5 min at 3,000 × *g*, and the water was discarded. Samples were resuspended with 200 µL PI staining solution, containing 1X PI (Propidium Iodide Flow Cytometry Kit, abcam, UK) and 0.1% triton in PBS 1X and incubated overnight at 4°C. Negative controls (no staining, or only FISH only samples) were incubated with the same staining solution without PI.

**Flow cytometry**

Samples were filtered through a 40-µm mesh size strainer (pluriSelect, Germany) and vortexed before processing with a DS CytoFLEX LX flow cytometer (Beckman Coulter Inc, USA) at a speed of 30 µL/min, using the CytExpert software. The gating procedure is outlined in **Figure S5A**. Symbiodiniaceae cells were selected using a forward scatter-height/side scatter-height (FSC-H/SSC-H) plot and singlets were gated on a FSC-H/FSC-Area plot. At least 20,000 single cells were processed for each replicate. PI signal was assessed using a 561 nm laser (emission 610 ± 10 nm) against FSC-W. The two distinct peaks were separated into G_1_ + G_0_ phases on one hand, and G_2_ + M phases on the other hand (signal twice as intense as G_1_+ G_0_). FISH signal was assessed using a 638 nm laser (emission 660 ± 5 nm) against FSC-H. Since no difference was measured between single-stain samples (FISH only, PI only) and double-stain samples (FISH + PI) for neither autofluorescence, FISH or PI signals **(Figure S5B)**, no compensation was applied and only the unstained and double-stained sub-samples of each sample were used for analysis. For each sample, the unstained sub-sample was used to determine cell autofluorescence in the FISH channel, and a quadrant was drawn to encompass unstained cells on one side (autofluorescence only), and stained cells on the other side (autofluorescence + potential FISH signal). The proportion of cells above the line was interpreted as the proportion of cells stained by FISH. This same quadrant was applied to the G_1_ + G_0_ and G_2_ + M portion of the sample, to assess the proportion of FISH-stained cells in each cell cycle phase. Considering the many low-speed centrifugations during the FISH experiment, we consider that any Symbiodiniaceae cell that exhibits signal stronger than autofluorescence bears closely associated bacteria (either intracellular or tightly attached to the outer cell surface). Results were analyzed with the FlowJo software. Statistical analyses and figures were performed in GraphPad. Normality was checked with a Shapiro-Wilk test and equal variances were assumed based on the equal number of samples in each condition.

**Cultures of bacteria closely associated with Symbiodiniaceae**

Symbiodiniaceae cultures **(Table S1)** cell counts were quantified (Life Technologies Countess II FL) and an aliquot of 10^6^ cells was pelleted, culture media removed and resuspended in 200 μL fRSS. Cells were twice washed by centrifugation at 5000 × *g* for 5 min with pelleted cells resuspended in 200 μL fRSS and brief vortexing between washings. Cells were finally resuspended in 200 μL fRSS. To separate the Symbiodiniaceae (ranging from 6-9 μm in diameter) and their closely associated (physically attached/intracellular) bacterial cells from loosely associated (planktonic) bacterial cells, the resuspended cells were filtered through a 5 μM mesh size strainer (pluriSelect, Germany) by centrifugation at 12,000 × *g* for 5 min. An inoculation loop was used to scrape Symbiodiniaceae cells and closely associated bacteria from the filter and spread plate inoculated onto three BD Difco™ Marine Agar 2216 (MA) and three Oxoid R2A Agar (R2A) prepared with fRSS, culture plates. Plates were incubated at 26°C for 7 d to facilitate bacterial colony growth. The morphology of bacterial colonies including form, elevation, margin, surface, texture, colour and opacity were recorded and representatives of all morphologies from plates with less than 300 colonies per plate were subcultured by the 16-streak method to attain culture purity. Pure cultures were stored in sterile 40% glycerol at -80^o^C.

**16S rRNA gene sequencing from cultured bacteria**

Individual pure freshly grown bacterial colonies were suspended in 20 μL sterile Milli-Q® water, incubated for 10 min at 95°C then used as templates in colony PCRs. PCR amplification of the bacterial 16S rRNA gene was with primers 27f and 1492r [70] in reactions containing 2 μL template DNA, 0.5 μL of each primer to a final concentration of 0.17 μM, 15 μL of 2x Mango Mix (Bioline 25034) and 12 μL of filter sterilised Milli-Q® water. The amplification cycle was: 5 min at 94°C; 30 cycles of 60 s at 94°C, 45 s at 50°C, and 90 s at 72°C; 10 min at 72°C; with a final holding temperature of 4°C. The PCR products were Sanger sequenced with primer 1492r at Macrogen (South Korea). Raw sequences were trimmed and proofread in Mega 7.0 (https://www.megasoftware.net/home).

**Phylogenetic tree construction**

Each individual 16S rRNA gene sequence was aligned using SILVA SINA alignment tool and the SILVA reference alignment [71]. The SILVA reference alignment searched the related sequences (nearest neighbours) to 95% min identity of the 16S rRNA gene sequences from this study, with an additional set of references from different bacterial phyla used to perform an alignment. The full alignment was stripped of columns containing 99% or more gaps, generating a final alignment containing 951 taxa (147 from this study) and 1,596 alignment positions. A maximum likelihood tree was inferred using RAxML-HPC BlackBox [72] as implemented on the CIPRES [73] web server under the GTRCAT evolutionary model. The RAxML inference included the calculation of 360 bootstrap iterations, with 100 randomly sampled to determine support values.


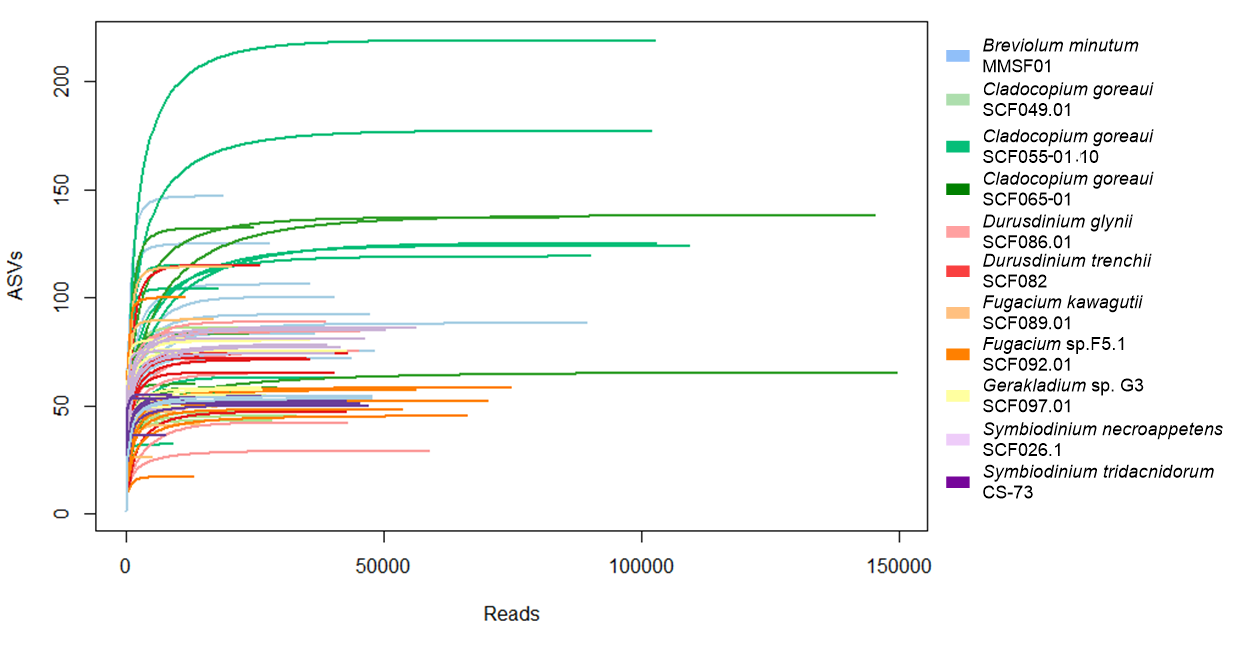


**Figure S1:** ASV rarefaction curves of 16S rRNA gene metabarcoding data, representing the diversity in all samples through observed richness.


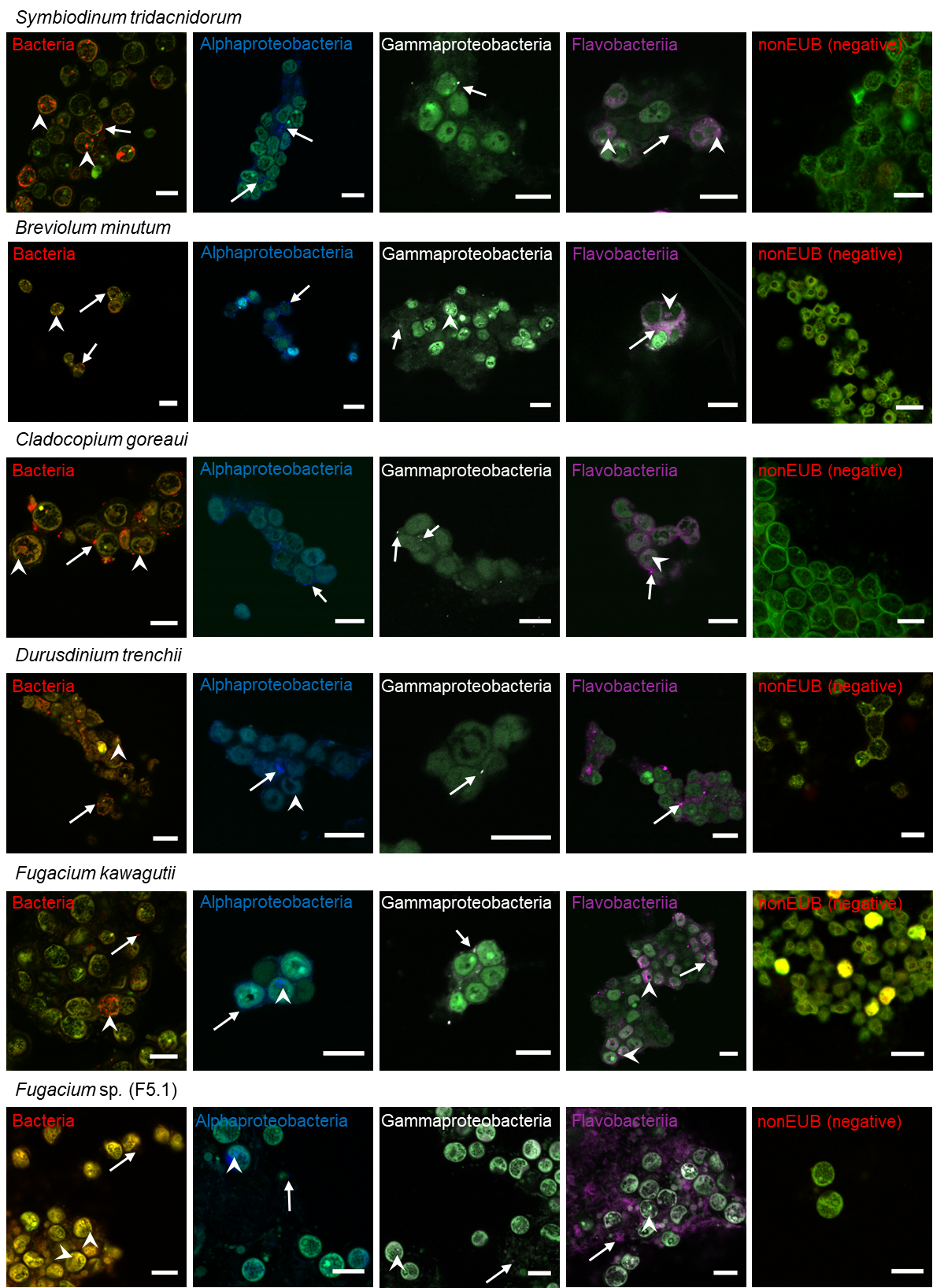


**Figure S2:** Localization of FISH-stained bacteria in six supplementary Symbiodiniaceae species, observed in confocal microscopy. Scale bar is 10 µm in all photos. Arrows point at extracellular bacteria and arrowheads point at intracellular bacteria. Green: Symbiodiniaceae; Red: EUB338-mix probe (all bacteria); Blue: Alf1B probe (Alphaproteobacteria); White: Gam42a probe (Gammaproteobacteria); Magenta: CF319 probe (Flavobacteriia).


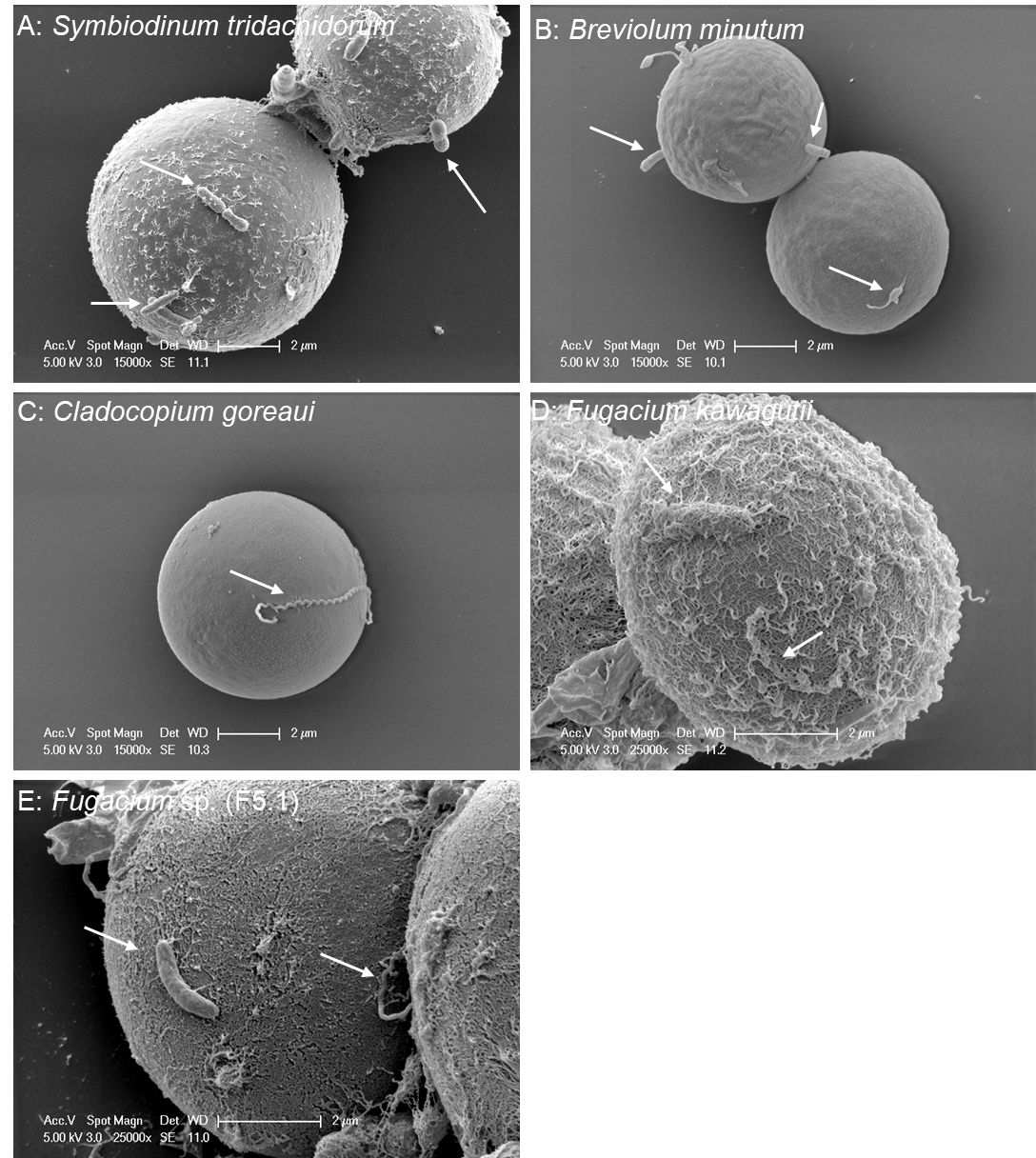


**Figure S3:** SEM photos of five Symbiodiniaceae species, highlighting the presence of bacteria attached to the cell wall. Arrows point at extracellular bacteria.

**
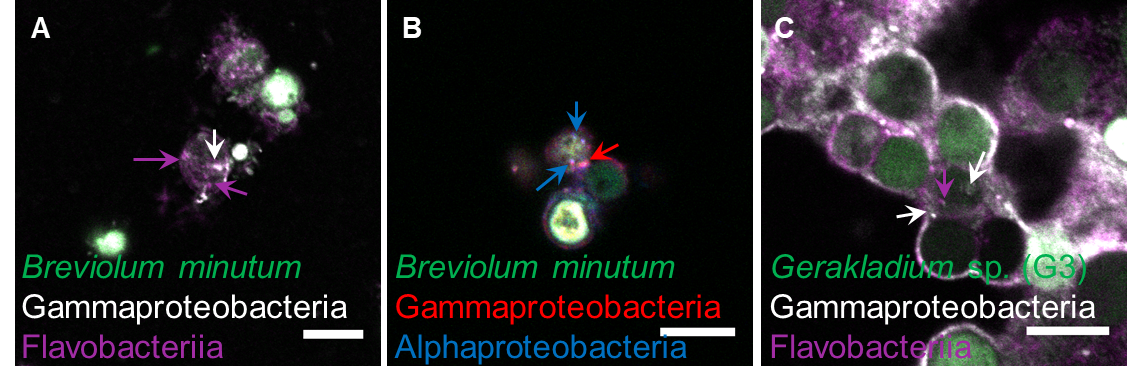
Figure S4:** Bacteria from different classes co-occur intracellularly in *Breviolum minutum* (A-B) and *Gerkladium* sp. (G3) (C). Bacteria were stained by FISH and samples were observed by CLSM. Scale bar is 10 µm in all photos. Green: Symbiodiniaceae; Blue: Alf1B probe (Alphaproteobacteria); White (A,C): Gam42a probe (Gammaproteobacteria); Red (B): Gam42a probe (Gammaproteobacteria); Magenta: CF319 probe (Flavobacteriia).


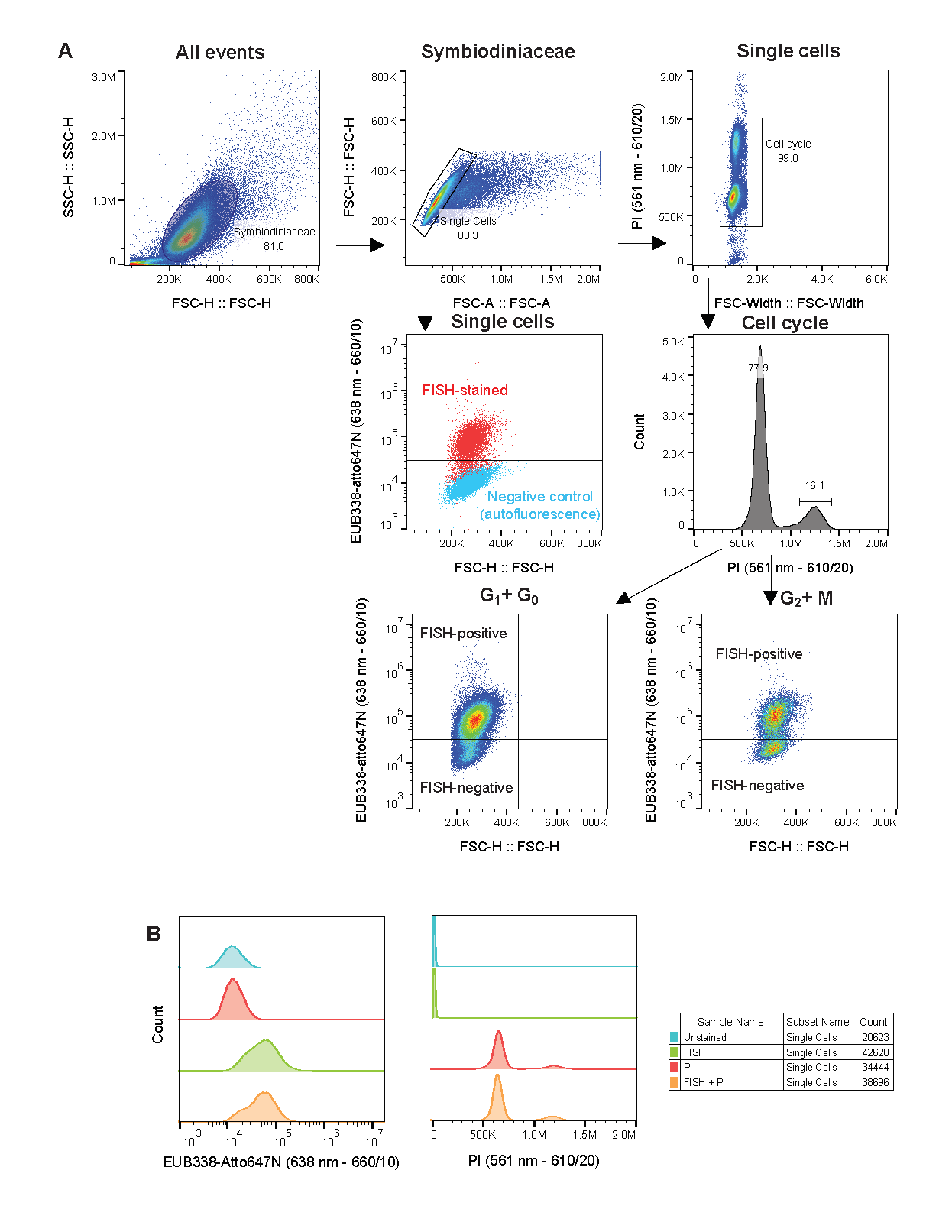


**Figure S5:** Procedure for flow cytometry acquisition. A: Gating procedure to isolate Symbiodiniaceae, single cells, and cells in G_1_ + G_0_ phase or in G_2_ + M phase, and to estimate the proportion of cells stained by FISH in all cells, cells in G_1_ + G_0_ phase, or cells in G_2_ + M phase. B: Comparison of FISH signal (left panel) and PI signal (right panel) in unstained, single-stained (FISH only or PI only), and double-stained (FISH and PI) sub-samples, showing that PI and FISH signals do not interfere with each other.


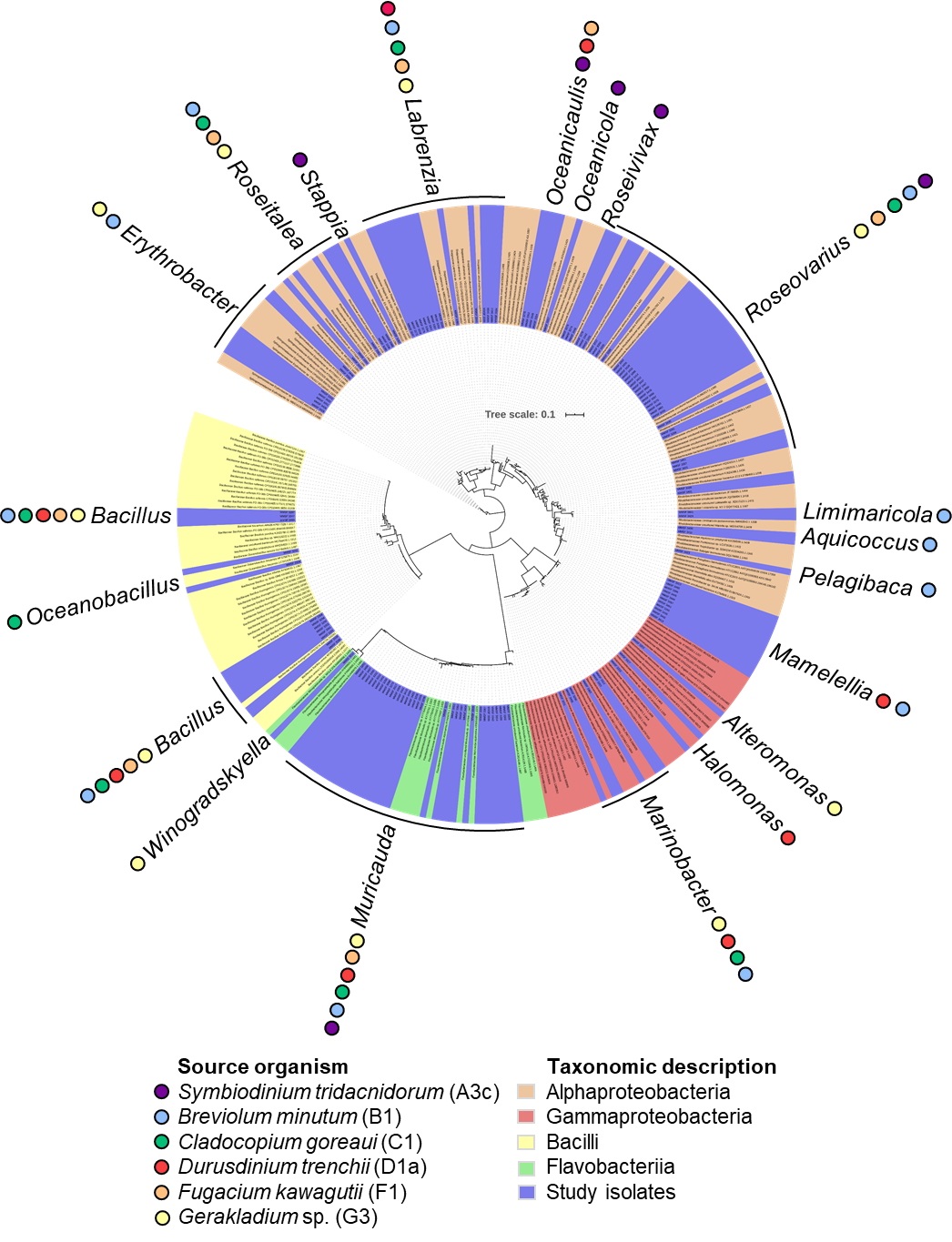


**Figure S6:** Maximum likelihood phylogenetic tree of the 16S rRNA gene placing the pure-culturable Symbiodiniaceae bacterial symbionts within the Bacteria domain. The final alignment contains 141 sequences from this study and was generated using the SILVA SINA alignment tool and the SILVA reference alignment. The tree was constructed using RAxML-HPC under the GTRCAT model of evolution.

**
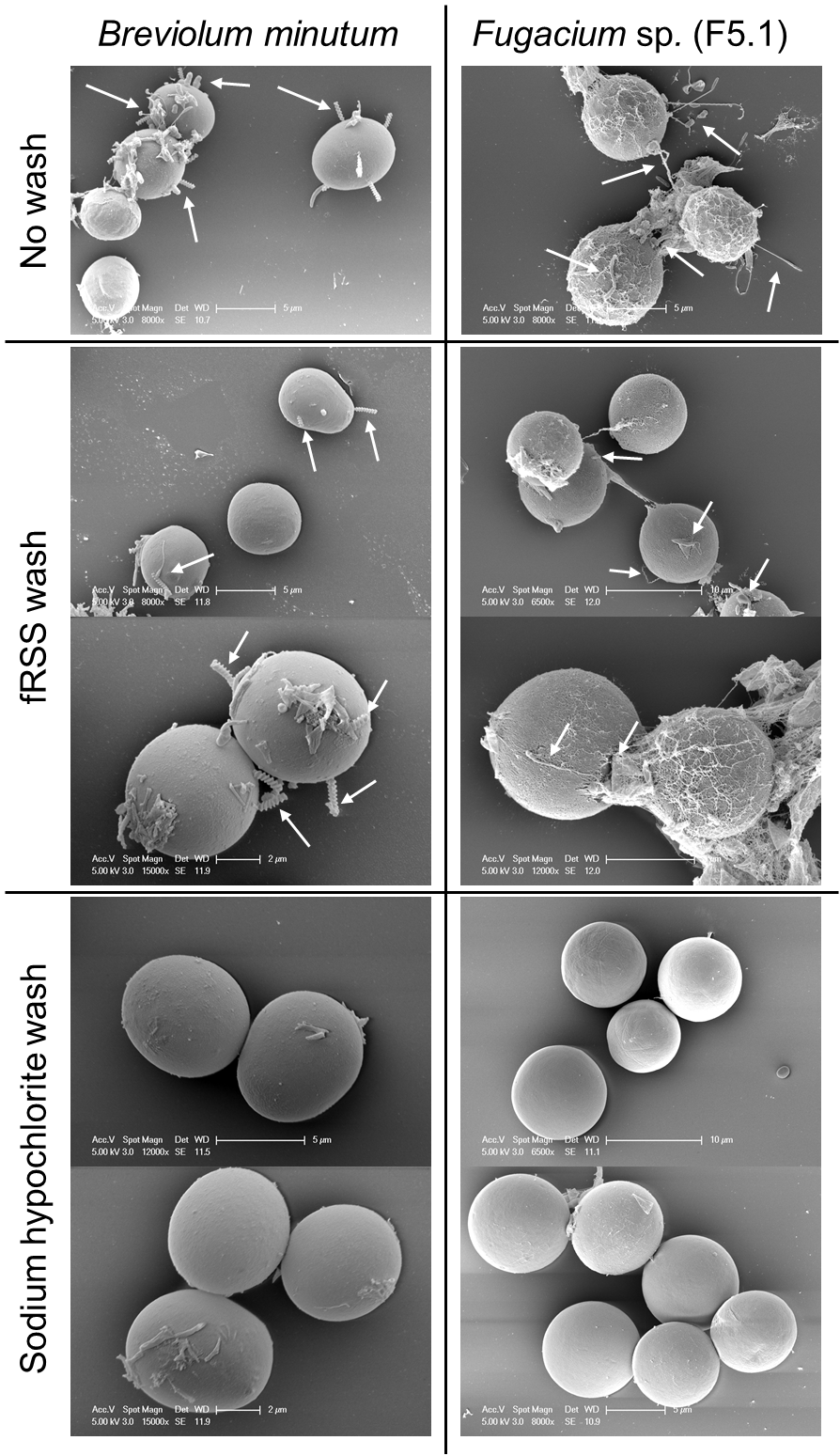
**

**Figure S7:** Efficient removal of extracellular bacteria through fRSS and bleach washes. *Breviolum minutum* and *Fugacium* sp*.* F5.1 were observed through SEM after no wash, one fRSS wash, or one sodium hypochlorite (6%) wash.

**
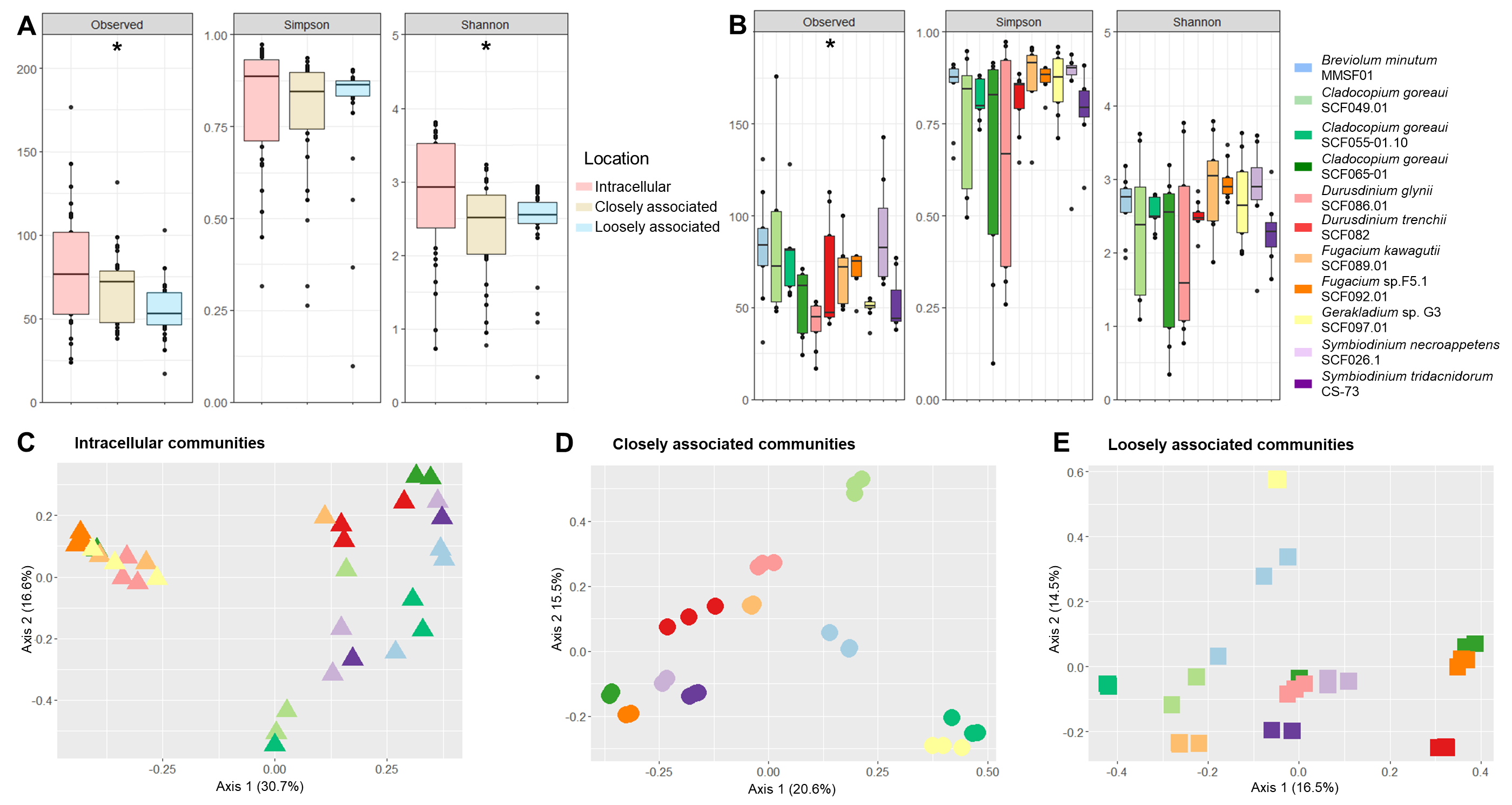
**

**Figure S8:** Alpha- and Beta-diversity metrics of the bacterial communities associated with Symbiodiniaceae cultures. A-B: Observed ASVs, Simpson index and Shannon’s index depending on location (A) or Symbiodiniaceae strain (B). Boxes represent the first and third quartiles for three independent replicates, the bar represents the median, and dots represent minimum and maximum values. Asterisks represent a significant effect (p < 0.05) of location (A) or Symbiodiniaceae strain (B) on the diversity metric, based on Kruskal-Wallis tests. C-E: PCoA visualization of beta-diversity of the bacterial communities independently in each of the three tested location in 11 Symbiodiniaceae strains and three locations, based on Bray-Curtis dissimilarity matrices. Each point is an individual sample. In each location, Symbiodiniaceae strain had significant effects based on PERMANOVA testing.


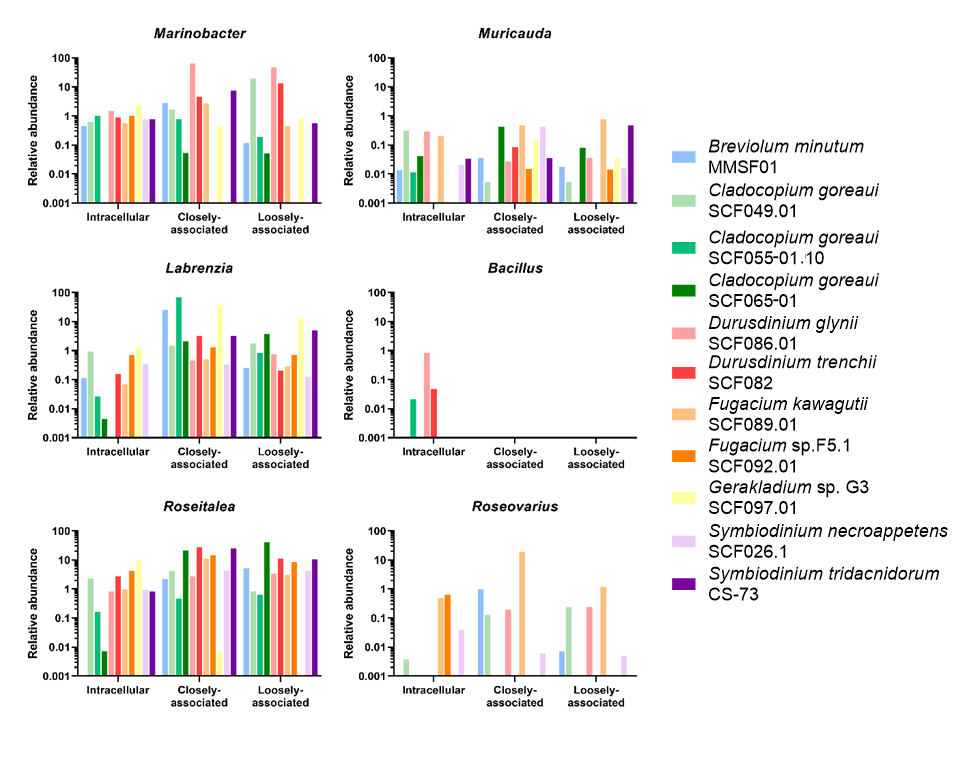


**Figure S9**: Relative abundance of six genera cultured from four or more Symbiodiniaceae species (see Figure 4) in the 16S rRNA gene metabarcoding data. Y-axis is a log scale.

**Table S1:** List of Symbiodiniaceae cultures used in this study and in each experiment. FISH: Fluorescence *in situ* Hybridization; SEM: Scanning Electron Microscopy.

**Table S2:** List of oligonucleotides probes used for Fluorescence *in situ* Hybridization.

*When GAM42a and CF319 were used simultaneously, 25% formamide was used.

**Supplementary Material Legends (provided separately as excel files):**

**Table S3:** List of contaminants identified in the 16S rRNA gene metabarcoding data, and their abundance in Symbiodiniaceae samples.

**Table S4:** Sum of relative abundances of intracellular, closely associated, and loosely associated core genera.

**Dataset S1:** Database of bacteria isolated from six species of Symbiodiniaceae. For each isolate: source Symbiodiniaceae species, morphology, closest BLAST result and full taxonomy. When there were several BLAST results with identical scores, the species column was left blank.

**Dataset S2:** Relative abundance of ASVs belonging to the Simkaniaceae family in all Symbiodiniaceae samples.

**Dataset S3:** Relative abundances of intracellular (A), closely associated (B), and loosely associated (C) core genera in all Symbiodiniaceae samples. A core genus is a genus that is present in every Symbiodiniaceae species within a given location.
